# Intensification of Photobiocatalytic Decarboxylation of Fatty Acids for the Production of Biodiesel

**DOI:** 10.1002/cssc.202002957

**Published:** 2021-02-02

**Authors:** Hong T. Duong, Yinqi Wu, Alexander Sutor, Bastien O. Burek, Frank Hollmann, Jonathan Z. Bloh

**Affiliations:** ^1^ Chemical Technology Group DECHEMA Research Institute Theodor-Heuss-Allee 25 60486 Frankfurt am Main Germany; ^2^ Department of Biotechnology Delft University of Technology van der Maasweg 9 2629HX Delft The Netherlands; ^3^ Department Institute of Measurement and Sensor Technology UMIT – University for Health Sciences, Medical Informatics and Technology GmbH Eduard-Wallnöfer-Zentrum 1, 1 6060 Hall in Tirol Austria

**Keywords:** biocatalysis, decarboxylation, photocatalysis, renewable fuels, scale-up

## Abstract

Light‐driven biocatalytic processes are notoriously hampered by poor penetration of light into the turbid reaction media. In this study, wirelessly powered light‐emitting diodes are found to represent an efficient and scalable approach for process intensification of the photobiosynthetic production of diesel alkanes from renewable fatty acids.

## Graphical abstract

**Setting light to fatty acids**: The photodecarboxylation of fatty acids to alkanes presents an intriguing way of producing biodiesel. Using delocalized wirelessly powered internal illumination leads to a reaction that is more efficient, dramatically accelerated, and readily scalable, clearly pointing towards industrial implementation.

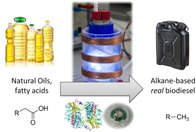

Biodiesel is an important pillar of the ongoing global transition from fossil energy carriers to renewable alternatives.^[1]^ Most widespread are fatty acid methyl esters (FAMEs) derived from natural fatty acids. FAMEs, however, exhibit some intrinsic disadvantages, which limit their application as drop‐in‐substitute for established fossil diesel. Due to their chemical nature as esters, the caloric value of FAMEs (ca. 32.6 MJ L^−1^) is significantly lower (by approx. 11 %) compared to fossil diesel (being essentially alkanes).^[2]^ Another drawback of FAMEs lies in their production by transesterification of natural fats and oils. Base‐ or acid‐catalyzed transesterification with methanol is an equilibrium reaction and therefore necessitates considerable molar surpluses of methanol to shift the equilibrium in the desired direction. Furthermore, free fatty acids present in natural feedstock have to be dealt with in order to minimize catalyst inactivation.

A very promising solution circumventing the above‐mentioned challenges is to simply decarboxylate fatty acids into the corresponding (C1‐shortened) alkanes (Scheme 1). The resulting biodiesel product chemically resembles fossil diesel and can be obtained from the starting material without further reagents in an irreversible reaction. Chemical catalysts facilitating the decarboxylation of (fatty)acids require relatively harsh reaction conditions^[3]^ thereby negatively influencing the energy consumption for biodiesel production and also leading to undesired side reactions.

**Scheme 1:**
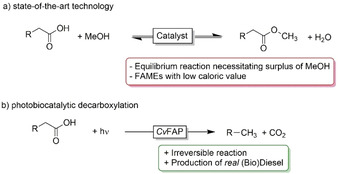
Comparison of traditional biodiesel synthesis by (trans)esterification (a) and the proposed photobiocatalytic decarboxylation (b).

Therefore, we became very interested in the recently reported photoactivated fatty acid decarboxylase from *Chlorella variabilis* NC64A (*Cv*FAP).^[4]^ Upon illumination with visible light (*λ*=450 nm) *Cv*FAP catalyzes the decarboxylation of a broad range of fatty acids.[^4^, ^5^, ^6^, ^7^, ^8^, ^9^, ^10^, ^11^, ^12^, ^13^, ^14^, ^15^] However, photochemical processes are severely limited by current reactor designs involving external illumination. Particularly with heterogeneous, optically nontransparent, and highly reflective reaction mixtures, the poor penetration depth of photons into the reactor leaves the majority of the contained catalysts unilluminated and therefore idle. This is particularly pronounced when scaling to larger reactor dimensions. As a result, the productivities of the photoenzymatic decarboxylation reactions generally lie in the range of a few mmol L^−1^ h^−1^ and therefore are orders of magnitude too low to be of industrial relevance.

To alleviate this shortcoming, we have recently established a new photoreactor concept comprising internal illumination by means of wirelessly powered light emitters (WLEs).^[16]^ This concept allows for high specific light intensities and is readily scalable. The WLEs are small (1 cm diameter) spherical polymer shells containing an LED and a receiving circuit for their electromagnetic energy supply (Figure 1). They can move freely inside the reaction medium and can be fluidized by gas flow or stirring. The power transfer is realized contactless by resonant inductive coupling (RIC) from coils mounted on the outside of the reactor (for more details, see the Supporting Information).[^17^, ^18^, ^19^]

**Figure 1:**
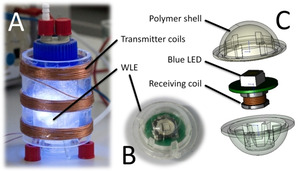
A photograph of the reactor setup employed in this study (A) as well as the individual wireless light emitters (WLE; B) and an explosion scheme of their makeup^[20]^ (C).

RIC‐powered LEDs have previously been used for algae cultivation and water purification,[^21^, ^22^, ^23^] but with much lower specific light intensities. Compared to earlier studies^[16]^ we have succeeded in improving the system resulting in much higher (ca. 27‐fold) specific light intensities. Therefore, we decided to apply the WLE concept to the photocatalytic decarboxylation of fatty acids to show that this technique is a step towards industrial application of photon‐driven biodiesel production.

To establish the proof‐of‐concept, we used the *Cv*FAP‐catalyzed decarboxylation of hexadecanoic acid (palmitic acid) into pentadecane. As the biocatalyst, we chose *Cv*FAP heterologously expressed in *Escherichia coli*. The whole cells harvested from the fermentation were used as the biocatalyst. It should be noted that *E. coli* cells not containing the expression vector for *Cv*FAP (under otherwise identical conditions) exhibited no detectable formation of pentadecane.

By using the conventional external illumination setup with conventional LEDs, palmitic acid was converted into pentadecane in approximately 90 % yield within 8 h (Figure 2). However, performing the same experiment using the proposed internal illumination resulted in full conversion in less than 20 min (Figure 2), corresponding to a more than 22‐fold acceleration of the product formation rate.

**Figure 2:**
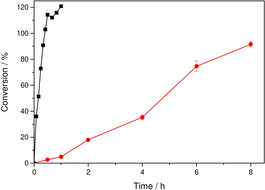
Photoenzymatic conversion of palmitic acid into pentadecane using external illumination (•) and internal illumination using WLEs (▪). Conditions: [palmitic acid]_0_=13 mM; [rec. *E. coli*]=50 g L^−1^ ([*Cv*FAP]=6 μM); buffer: 100 mM Tris‐HCl (pH 8.5), 30 % v/v DMSO, volume=50 mL; *T*=20 °C; photon flux of external illumination: 27 mmol L^−1^ h^−1^ and of internal illumination: 390 mmol L^−1^ h^−1^ (40 WLEs).

Admittedly, most of this rate acceleration is due to the higher light intensity enabled by the internal illumination technique. However, even a direct comparison of internal and external illumination at the same incident photon flux density revealed a clear advantage of the former with about 1.8‐fold higher observed reaction rates (see below).

Using whole recombinant *E. coli* cells generally resulted in the accumulation of approximately 15–16 mmol L^−1^ of pentadecane (ca. 120 % yield). Control experiments in the absence of palmitic acid revealed that the additional product originated from conversion of cell membrane components of the whole‐cell catalyst, thereby explaining the higher conversion (Figure 2).

Encouraged by the impressive rate‐acceleration, we next systematically investigated the influence of catalyst loading and light intensity on the productivity of the photoenzymatic decarboxylation reaction. At a fixed light intensity, the product formation rate steadily increased with increasing catalyst concentration (Figure 3). At low enzyme concentration, the product formation rate increased approximately linearly just like for an ordinary enzyme. Here, the enzyme appears to be oversaturated by photons and is only limited by its own, intrinsic catalytic activity. Indeed, the maximum TOF (TOF=mol_product_×*t* ^−1^×mol_*Cv*FAP_^−1^) observed in these experiments was 4.0 s^−1^, which is an order of magnitude higher than previously reported.[^4^, ^15^] At higher enzyme concentration, this was no longer the case and gradually, photon limitation manifested resulting in a plateau where the rate no longer increases.

**Figure 3:**
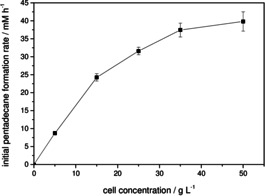
Biocatalyst‐concentration dependency of the photoenzymatic decarboxylation of palmitic acid to pentadecane. Conditions: [palmitic acid]_0_=13 mM; [rec. *E. coli*]=50 g L^−1^ ([*Cv*FAP]=6 μM); buffer: 100 mM Tris‐HCl (pH 8.5), 30 % v/v DMSO, volume=50 mL; *T*=20 °C; internal illumination with a photon flux of 390 mmol L^−1^ h^−1^ (40 WLEs).

Similarly, also the light intensity directly influenced the overall reaction rate (Figure 4). After an initial almost linear increase, mild light saturation effects became apparent at higher photon flux density resulting in a slightly diminished response. This is a typical behavior also often observed for heterogeneous photocatalytic reactions.^[24]^ It is an inevitable consequence of the exponential light intensity decay as at higher light intensity, the areas near the light source will start to show symptoms of photon saturation while farther away, this is still far from happening. As mentioned above, the external illumination experiments show the same trend, albeit with systematically lower rates. It also appears that in this case, the reaction rate levels off at the lower maximum value, although this could not be experimentally verified yet as no light source strong enough was available.

**Figure 4:**
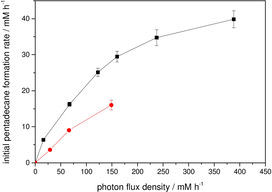
Comparison of the initial pentadecane formation rate using WLEs for internal illumination (▪) and a LED for external illumination (•) at different light intensities. Conditions: [palmitic acid]_0_=13 mM; [rec. *E. coli*]=50 g L^−1^ ([*Cv*FAP]=6 μM); buffer: 100 mM Tris‐HCl (pH 8.5), 30 % v/v DMSO, volume=50 mL; *T*=20 °C. The variation of the internal illumination light intensity was achieved by addition of different numbers of WLEs while for external illumination this was realized by changing the driver current.

The advantage of the internal illumination can be attributed to two effects. First of all, it is less affected by reflection losses resulting from the strongly turbid reaction suspension, as more light is productively scattered back into the medium rather than out of the reactor. Additionally, the light is also more evenly distributed throughout the reactor, avoiding “bright” spots and the associated local photon oversaturation.^[24]^ We expect that the latter effect and with it the advantage of internal illumination will become even more pronounced when scaled to larger reactor volumes due to the then reduced (illuminated) surface‐to‐volume ratio in case of external illumination. To the best of our knowledge, this is the first time that such a significant advantage of internal illumination has been experimentally demonstrated.

In line with earlier estimations, the observed apparent quantum yield (AQY) of the reaction is remarkably high with up to 39.8 % (for details, see the Supporting Information).^[4]^ Interestingly, the decarboxylation reaction is endothermic with a heat of reaction of +71.1 kJ mol^−1^ (calculated from heats of combustion; see the Supporting Information). The thermodynamic driving force stems from the photons used with an energy of 265.9 kJ mol^−1^ (for *λ*=450 nm). This also means that the reaction is actually photosynthetic, i. e., part of the photon energy is found in the product, making the high AQY even more remarkable as such reactions typically suffer from poor efficiency.^[25]^

At perfect quantum efficiency and assuming loss‐free photon generation, the maximum theoretical energy efficiency of the reaction [Δ_c_ *H* ^0^_products_×(Δ_c_ *H* ^0^_educts_+*E*_photons_)^−1^] would be as high as 98.1 %. Even though the actually achieved energy efficiency in this non‐optimized lab setup was 32.1 %, already 89.8 % appear possible with the AQY reached here by optimizing the setup to match state‐of‐the‐art efficiencies for the electronic parts and the inductively coupled energy transfer^[26]^ as well as the LEDs (see the Supporting Information).[^27^, ^28^] This is remarkable considering that established processes for the synthesis of renewable fuels such as Power‐to‐Liquids reach energy efficiencies around or below 50 %.^[29]^

Using homogeneously dissolved fatty acids does not represent a scalable approach for the large‐scale transformation of fatty acids into alkanes due to the low product titers. We therefore also investigated a two liquid phase approach to increase the overall payload of the fatty acids in the reactor system. For this, we chose triolein as the organic phase (representing future oil phases) to form a 200 mM solution of palmitic acid. More than 50 % conversion was achieved within 2 h with a rate similar to the one achieved in the monophasic system. However, at this point the product formation abruptly ceased (see the Supporting Information, Figure S6). Further addition of fresh catalyst resulted in further product formation. This experiment highlights the current limitation of the proposed photobiocatalytic alkane production system being the comparably poor long‐term stability of the enzyme catalyst under process conditions. In our experiments the turnover numbers for *Cv*FAP (TON=mol_product_×mol_*Cv*FAP_^−1^) never exceeded 9.000. These turnover numbers are well in the range of TONs previously observed for *Cv*FAP[^10^, ^11^, ^12^, ^13^, ^14^, ^15^] indicating that the light intensity itself is not the main parameter for *Cv*FAP inactivation. This supports the *Cv*FAP inactivation mechanism proposed by Scrutton and co‐workers, assuming that intermediate radical species occurring in the catalytic mechanism may cause inactivation of the biocatalyst.^[6]^

In the present study, we have demonstrated that the rate of photobiocatalytic reactions, such as the decarboxylation of fatty acids, can be dramatically increased by using intensified internal illumination. The same technique also allows to seamlessly scale‐up the production volume to industrially relevant dimensions. The conditions presented herein seem to approach the limit for wild type *Cv*FAP. Already, (photon) saturation effects become apparent which probably make further intensification challenging. Nevertheless, at the productivity achieved in this study, the process could produce 264 mL of pentadecane per liter of reaction volume each day, clearly pointing towards larger scale implementation. This study, however, also revealed a current shortcoming of the proposed photosynthetic fuel generation being the rather low operational stability of *Cv*FAP, which needs further improvement. The low turnover numbers mean that the enzyme needs to be continuously replenished, resulting in a high cost contribution of the biocatalyst.^[30]^ On one hand, current expression levels of *Cv*FAP (ca. 10 % of the total protein) are still comparably low,^[31]^ necessitating relatively high loadings of *E. coli* cells in the reaction. Optimization of the expression conditions will alleviate this issue. On the other hand, we are convinced that further improvements can be expected from fermentation optimization and protein engineering resulting in more robust enzyme variants to render the envisioned photosynthetic production of alkanes economically feasible.

## Conflict of interest

The authors declare no conflict of interest.

## Supporting information

As a service to our authors and readers, this journal provides supporting information supplied by the authors. Such materials are peer reviewed and may be re‐organized for online delivery, but are not copy‐edited or typeset. Technical support issues arising from supporting information (other than missing files) should be addressed to the authors.

Supplementary
