# Supplementary material for: Intensification of Photobiocatalytic Decarboxylation of Fatty Acids for the Production of Biodiesel
Source: ChemSusChem. 2021 Feb 2;14(4):1053–6. doi: 10.1002/cssc.202002957 (PMC7986711; doi:10.1002/cssc.202002957)
Supplement: Supplementary file 1 — Supplementary [file CSSC-14-1053-s001.pdf]

ChemSusChem

Supporting Information

Intensification of Photobiocatalytic Decarboxylation of Fatty Acids for the Production of Biodiesel

Hong T. Duong+, Yinqi Wu+, Alexander Sutor, Bastien O. Burek,* Frank Hollmann,* and Jonathan Z. Bloh*© 2021 The Authors. ChemSusChem published by Wiley-VCH GmbH. This is an open access article under the terms of the Creative Commons Attribution License, which permits use, distribution and reproduction in any medium, provided the original work is properly cited.

Supporting Information

Intensification of photobiocatalytic decarboxylation of fatty acids for the production of biodiesel

H. T. Duong,[a‡] Y. Wu,[b‡] A. Sutor,[c] B. O. Burek,[a*] F. Hollmann,[b*] J. Z. Bloh[a*]

[a] H. T. Duong, Dr. J. Z. Bloh, Dr. B. O. Burek

Chemical Technology Group

DECHEMA Research Institute

Theodor-Heuss-Allee 25, 60486 Frankfurt am Main (Germany)

E-mail: jonathan.bloh@dechema.de, bastien.burek@dechema.de

[b] Y. Wu, Dr. F. Hollmann

Department of Biotechnology

Delft University of technology

van der Maasweg 9, 2629HX Delft (The Netherlands) E-Mail: f.hollmann@tudelft.nl

[c] Univ.-Prof. Dr. A. Sutor

Department Institute of Measurement and Sensor Technology

UMIT - University for Health Sciences, Medical Informatics and Technology GmbH

Eduard-Wallnöfer-Zentrum 1, 16060 Hall in Tirol (Austria)

Table of Contents

1. **Experimental Part** **S3**
   1.1 **Photocatalytic setups** **S3**
   1.2 **Chemical actinometry** **S4**
   1.3 **Photocatalytic pentadecane production with CvFAP@E.coli** **S4**
2. **Results and Discussion** **S5**
   2.1 **Determination of the photon flux density with chemical actinometry** **S5**
      2.1.1 External illumination S5
      2.1.2 Internal illumination S6
   2.2 **Pentadecane production with different cell concentrations** **S7**
   2.3 **Pentadecane production as a function of the number of WLEs** **S8**
   2.4 **Efficiency calculations** **S8**
   2.5 **Theoretical energy efficiency** **S9**
   2.6 **Two liquid phase reaction** **S10**
3. **Notes and references** **S10**
4. **Author Contributions** **S11**

1. Experimental Part

1.1 Photocatalytic set-ups

The reactions were performed in a cylindrical glass reactor (100 mL, 56 mm diameter, 105 mm height). For internal illumination wireless light emitters (WLE) were used which consist of a ferrite core inductor (WE-PD2 4532 10 µH, Würth), a capacitor (82 nF) and a blue LED (XPGDRY-L1-0000-00401, 451 nm peak emission, Cree Inc.), connected all in parallel. These elements form an oscillating circuit with a certain resonance frequency and are encapsulated in a SiO_2 coated cyclic olefin polymer hollow with a diameter of 10 mm. Preliminary testing showed that the WLE shell was fully compatible with the employed solvents, there was no detectably polymer dissolution in the reaction mixture before or after the reaction. To power the WLEs, the reactor was surrounded with three copper wire coils (18 turns each) in series with a distance of 20 mm, mounted on a PMMA tube (75 mm diameter, 105 mm height). The coil was connected to a capacitor with an appropriate capacitance to generate a series resonant circuit with a resonance frequency of 178 kHz. This circuit was connected in parallel to an oscillator amplifier circuit with the same resonant frequency of 178 kHz. The reaction mixture was stirred with a PEEK stirrer with a PTFE 4-blade radial flow impeller. Further details of the reactor have been described earlier by us.[1] For external illumination a collimated blue LED (M450LP1, 450 nm peak emission, Thorlabs) has been used.

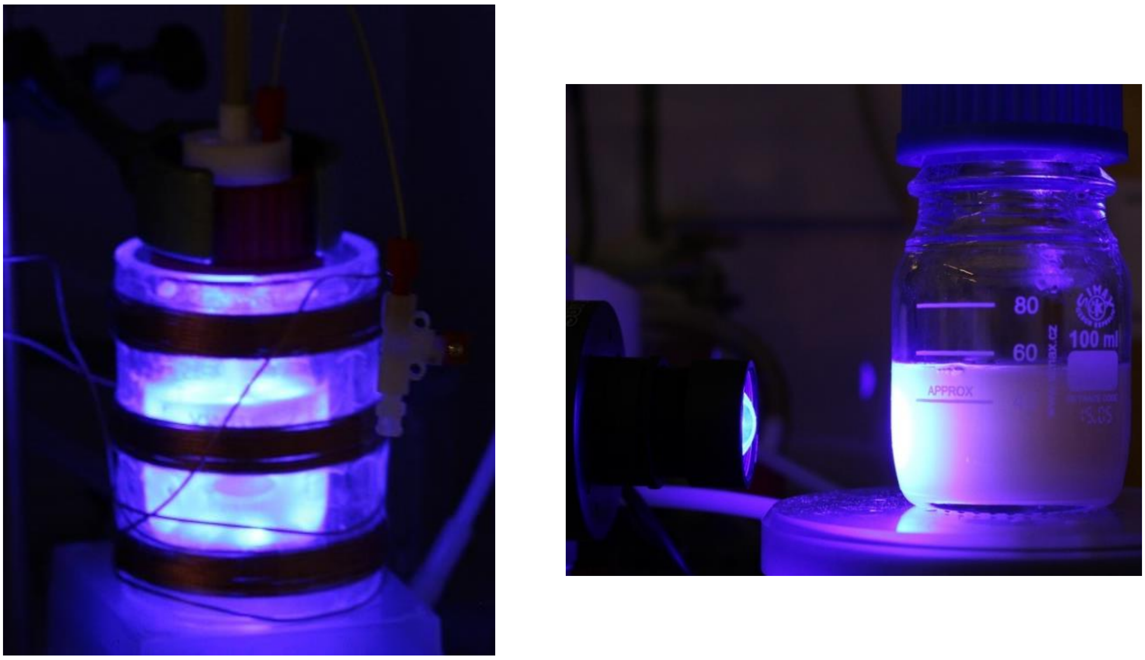

Figure S1. Pictures of the internal (left) and external (right) illuminated reactors under operating conditions.

1.2 Chemical actinometry

The volumetric photon flux density (q_P) was determined by means of ferrioxalate actinometry according to Hatchard and Parker.[2] 50 mL of a freshly prepared ferrioxalate solution (37.5 mM in 0.05 M H_2SO_4 (98% Merck)) was added to the reactor and irradiated either with a varying amount of WLE or the external blue LED. At defined intervals, 25 µL samples of the irradiated ferrioxalate solution were taken and mixed with 175 µL of a solution consisting of 7.5 mL H_2SO_4 (0.05 M), 2 mL 0.1% 1,10-phenantroline (Carl Roth) solution, 5 mL sodium acetate solution (1M (Carl Roth)) and 3 mL deionized water. The absorbance of the resulting ferroin complex was measured at the maximum absorption of 510 nm using a UV-Vis microplate reader (PowerWave HT, BioTek). To quantify the concentration of Fe(II) calibration curves using Fe(SO_4) have been used. The photon flux was calculated subsequently using the iron(II) generation rate as well as the quantum yield and transmission of the ferrioxalate solution. The power consumption was measured at the wall plug using a commercial power meter (KD-302, profitec).

1.3 Photocatalytic pentadecane production with *Cv*FAP@E.coli

*Cv*FAP@*E.coli* were added to 35 mL of TRIS-HCl buffer (100 mM, pH= 8.5) and properly mixed. The mixture was added to the photoreactor and filled up to 50 mL total volume with palmitic acid (43.3 mM, Sigma Aldrich) dissolved in DMSO (> 99.7 %, Sigma Aldrich). 400 µL samples were taken from the suspension at defined intervals and mixed with 100 µL of HCl (37%, Carl Roth) in an ultrasonic bath for 10 minutes. Afterwards the samples were extracted with 250 µL ethyl acetate (>99.5%, Roth). The organic phase was dried with $MgSO_4$ (VWR) and analyzed *via* GC-MS (GCMS-QP5050, SHIMADZU, column: Rxi-5HT, Restek GmbH, 30 m length, 0.1 µm film thickness, 0.25 mm ID, max. temperature 400°C, method: He carrier, 30 kPa constant pressure, split (split ratio: 21), start: 70 °C for 2 min, with a heating rate of 25 °C min^{-1} up to 300°C, total time: 13 min. MS settings: Acquisition Mode: Scan, Interface Temp.: 280 °C, Solvent Cut Time: 2.5 min, Detector Voltage: -50 kV, Threshold: 1000, Interval: 0.5 sec, start time: 2.5 min, end time: 11.03 min, start m/z: 10, end m/z: 350, scan speed: 1000, Ion Source: EI)

2. Results and Discussion

2.1 Determination of the photon flux density with chemical actinometry

2.1.1 External illumination

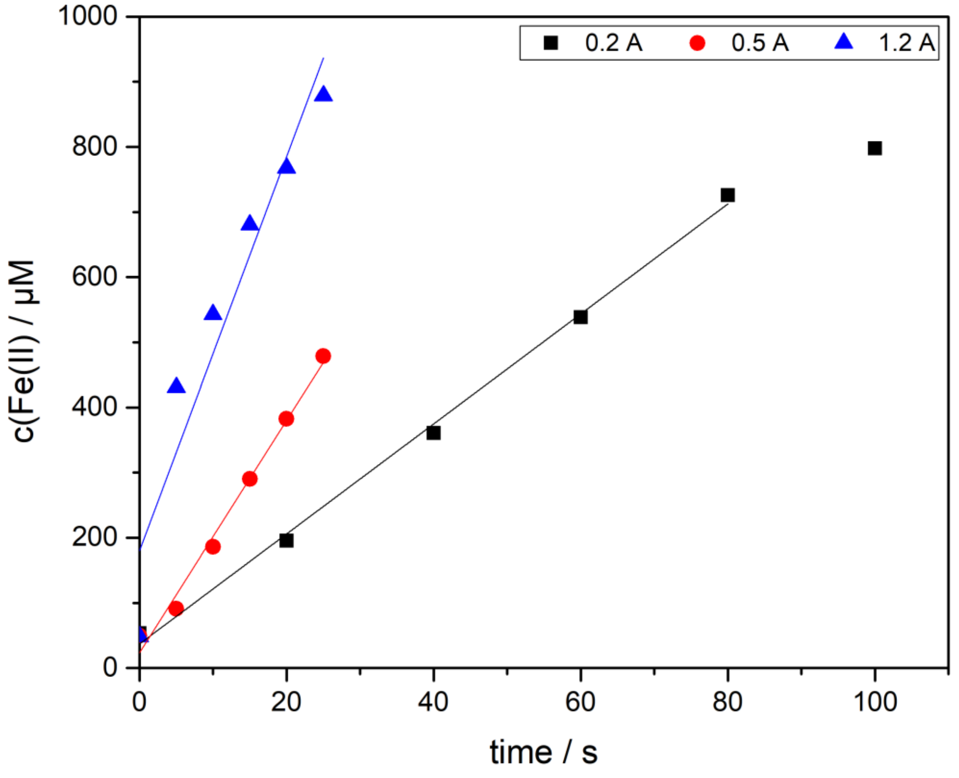

Figure S2. Time courses of the iron(II) generation under illumination of the ferrioxalate solution. Reaction conditions: 50 mL ferrioxalate solution, varying the applied current to the LED from 0.2 to 1.2 A, under Ar atmosphere.

Table S1. Determined volumetric photon flux (q_P) in dependence of the applied LED current using external illumination.

LED current / A	q_P / mM h^{-1}
0.2	29.1
0.5	66.2
1.2	149.0

2.1.2 Internal illumination

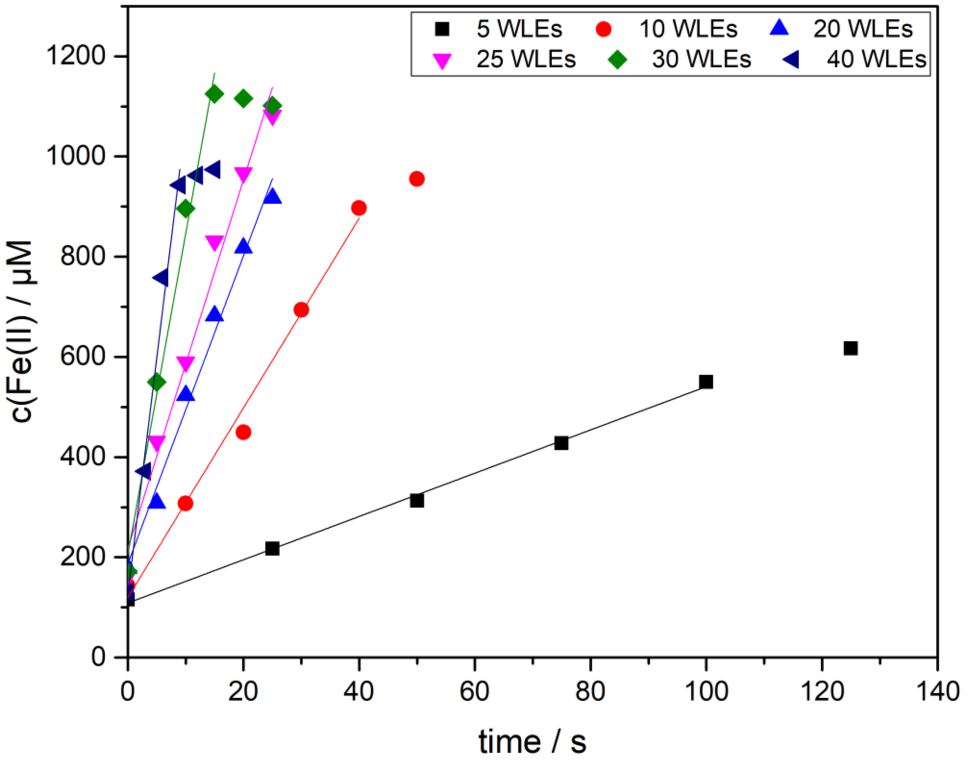

Figure S3. Time courses of the iron(II) generation under illumination of the ferrioxalate solution. Reaction conditions: 50 mL ferrioxalate solution, 5-40 WLE under Ar atmosphere.

The radiant flux in the reactor (P_{light}) can be calculated by multiplying the respective photon flux density (q_p) with the reaction volume (50 mL) and the molar energy of the photons (see eq. 1). The wall plug efficiency is the quotient of the light power and the electrical power consumption (P_{el}) at the wall plug (see eq. 2).

$$P_{light} = q_P \times V \times \frac{h \times c}{\lambda} \times N_A \qquad \text{(eq.1)}$$

$$WPE = \frac{P_{light}}{P_{el}} \qquad \text{(eq.2)}$$

Table S2. Determined photon flux (q_p) with the respective electrical power consumption at the wall plug (P_{el}) as well as light output (P_{light}) and the resulting wall plug efficiency (WPE) of the internal illumination.

N_{WLE}	Volume / mL	q_p / mE L^{-1} h^{-1}	P_{el} / W	P_{light} / mW	WPE / %
5	50	16.6	6.0	60	1.0
10	50	67.0	7.6	245	3.2
20	50	122.6	9.4	450	4.8
25	50	160.1	9.9	590	6.0
30	50	237.5	10.7	875	8.2
40	50	388.3	11.8	1430	12.1

2.2 Pentadecane production with different cell concentrations

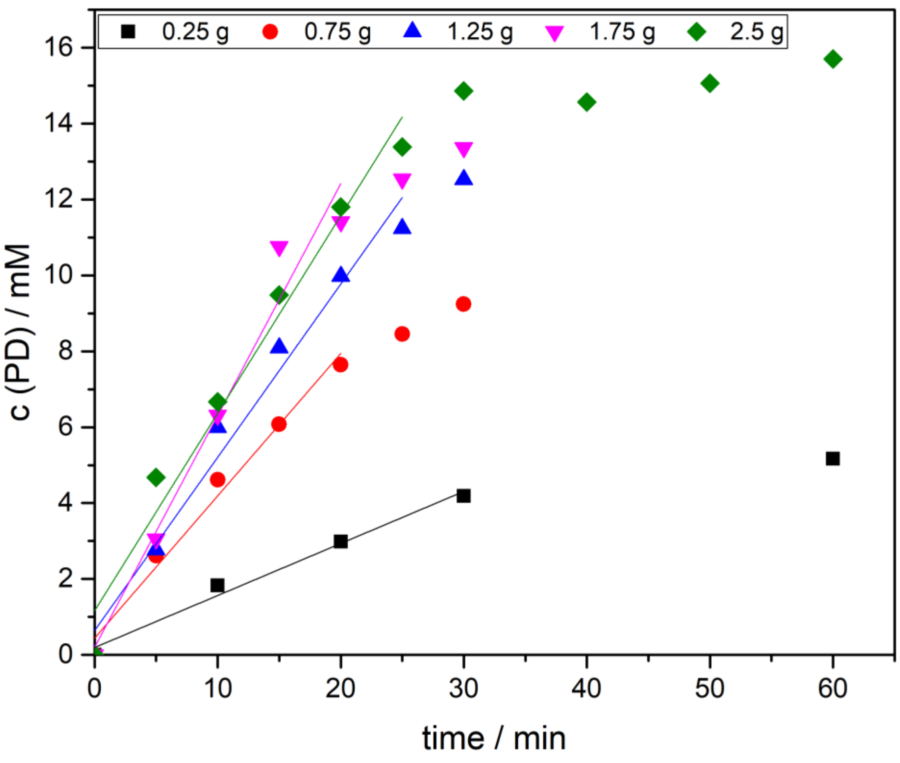

Figure S4. Concentration-time-profiles for the pentadecane production with different cell concentrations. Reaction conditions were as follows: 13.1 mM palmitic acid, 50 mL total volume, 100 mM TRIS-HCl buffer, varying the *Cv*FAP@*E.Coli* concentration from 0.25 to 2.5 g corresponding to respective enzyme concentrations of 0.6 to 6 µM, 40 WLE, 20 V amplifier voltage.

2.3 Pentadecane production as a function of the number of WLEs

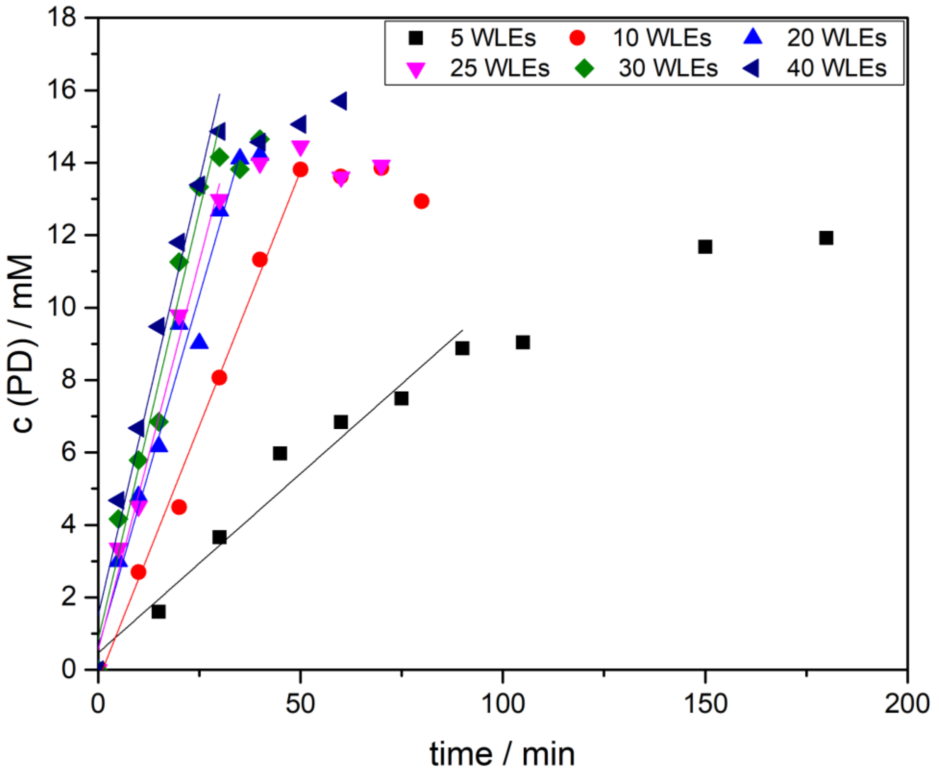

Figure S5. Concentration-time-profiles for the pentadecane production with different numbers of WLEs. Reaction conditions were as follows: 13.1 mM palmitic acid, 50 mL total volume, 100 mM TRIS-HCl buffer, 2.5g *Cv*FAP@*E.Coli*, varying the number of WLEs from 5 to 40, 20 V amplifier voltage.

2.4 Efficiency calculations

The apparent quantum yield (AQY) can be calculated by dividing the rate of pentadecane production (k_{PD}) by the photon flux density (q_P, see eq. 3).

$$AQY = \frac{k_{PD}}{q_P} \qquad \text{(eq.3)}$$

The overall energy efficiency (EE) can be calculated by dividing the combustion energy of the product pentadecane ($\Delta_c H_{PD}^0$ = -10048.7 kJ mol^{-1})[3] by the combustion energy of the educt palmitic acid ($\Delta_c H_{PA}^0$ = -9977.6 kJ mol^{-1})[4] plus the energy necessary to generate the photons (eq. 4). The former can be calculated according to eq. 5 from the molar photon energy divided by the efficiency of generating the photons (WPE) and the efficiency of converting a photon into the product (AQY).

$$EE = \frac{\Delta_c H^0_{PD}}{\Delta_c H^0_{PA} + E_{photons}} \quad \text{(eq.4)}$$

$$E_{photons} = \frac{\frac{h \times c}{\lambda} \times N_A}{WPE \times AQY} \quad \text{(eq.5)}$$

Table S3. Kinetic and efficiency data for Pentadecane production as a function of different number of WLEs.

N WLE	q_p / mE L^{-1} h^{-1}	k_{PD} / mM h^{-1}	AQY / %	EE / %
5	16.0	6.4	39.8	12.9
10	67.0	16.2	24.2	23.0
20	122.6	25.1	20.5	27.2
25	160.1	29.5	18.4	29.4
30	237.5	34.7	14.6	31.2
40	388.3	39.8	10.3	32.1

2.5 Theoretical energy efficiency

The presented system is non-optimized in terms of efficiency of amplifier, LED and resonant inductive coupling (RIC). Assuming literature known values of 91 % for the amplifier and 84 % for RIC, 80 % for a blue LED[5,6] and a transmission of 90 % through the polymer shell[1] of the WLE, the WPE of a state-of-the-art system could be as high as 55 %. With this WPE and the maximum AQY of 39.8 % observed in this work, a theoretical energy efficiency of 89.8 % for the conversion of palmitic acid into pentadecane can be realized.

2.6 Two liquid phase reaction

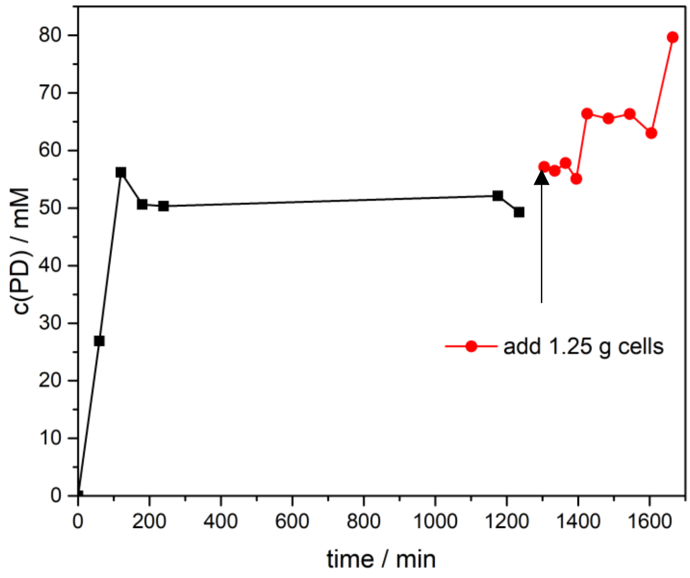

Figure S6. Concentration-time-profiles for the pentadecane production in a two liquid phase system using triolein as organic phase. Reaction conditions were as follows: 25 mL of 200 mM palmitic acid in triolein, 25 mL of a 100 mM TRIS-HCl buffer containing 2.5 g *Cv*FAP@*E.Coli*, after 1305 min 1.25 g of cells were added, 40 WLE, 20 V amplifier voltage.

3. Notes and references

1. B. O. Burek, A. Sutor, D. W. Bahnemann and J. Z. Bloh, *Catal. Sci. Technol.*, 2017, **7**, 4977–4983.
2. C. G. Hatchard and C. A. Parker, *Proc. R. Soc. A Math. Phys. Eng. Sci.*, 1956, **235**, 518–536.
3. E. J. Prosen and F. D. Rossini, *J. Res. Natl. Bur. Stand. (1934).*, 1945, **34**, 263.
4. H. A. Swain, L. S. Silbert and J. G. Miller, *J. Am. Chem. Soc.*, 1964, **86**, 2562–2566.
5. M. Auf der Maur, A. Pecchia, G. Penazzi, W. Rodrigues and A. Di Carlo, *Phys. Rev. Lett.*, 2016, **116**, 027401.
6. L. Y. Kuritzky, A. C. Espenlaub, B. P. Yonkee, C. D. Pynn, S. P. DenBaars, S. Nakamura, C. Weisbuch and J. S. Speck, *Opt. Express*, 2017, **25**, 30696.

4. Author Contributions

H.T.D., Y.W., F.H., J.Z.B. and B.O.B. designed the experiments.

H.T.D. determined the photon fluxes for the WLE reactor and for the external illumination.

H.T.D. and Y.W. carried out the photoenzymatic experiments

Y.W. carried out the fermentation of CvFAP, purified the enzyme and characterised the enzyme preparation.

H.T.D., Y.W., F.H., J.Z.B. and B.O.B. analyzed the data and co-wrote the manuscript.

A.S. designed the WLE's electronic circuit and the field generator for powering them.

F.H., J.Z.B. and B.O.B. supervised the project. F.H., J.Z.B. and B.O.B. conceived the concept.
